# Gravity models to classify commuting vs. resident workers. An application to the analysis of residential risk in a contaminated area

**DOI:** 10.1186/1476-072X-10-11

**Published:** 2011-01-27

**Authors:** Guido Signorino, Roberto Pasetto, Elisa Gatto, Massimo Mucciardi, Marina La Rocca, Pierpaolo Mudu

**Affiliations:** 1 Department of Economics, Statistics, Mathematics and Sociology, University of Messina, Via Tommaso Cannizzaro 278, Messina, Italy; 2 Department of Environment and Primary Prevention, Istituto Superiore di Sanità, Viale Regina Elena 299, Rome, Italy; 3 World Health Organization - European Centre for Environment and Health, Via Francesco Crispi 10, 00187 Rome, Italy

## Abstract

**Background:**

The analysis of risk for the population residing and/or working in contaminated areas raises the topic of commuting. In fact, especially in contaminated areas, commuting groups are likely to be subject to lower exposure than residents. Only very recently environmental epidemiology has started considering the role of commuting as a differential source of exposure in contaminated areas. In order to improve the categorization of groups, this paper applies a gravitational model to the analysis of residential risk for workers in the Gela petrochemical complex, which began life in the early 60s in the municipality of Gela (Sicily, Italy) and is the main source of industrial pollution in the local area.

**Results:**

A logistic regression model is implemented to measure the capacity of Gela "central location" to attract commuting flows from other sites. Drawing from gravity models, the proposed methodology: a) defines the probability of finding commuters from municipalities outside Gela as a function of the origin's "economic mass" and of its distance from each destination; b) establishes "commuting thresholds" relative to the origin's mass. The analysis includes 367 out of the 390 Sicilian municipalities. Results are applied to define "commuters" and "residents" within the cohort of petrochemical workers. The study population is composed of 5,627 workers. Different categories of residence in Gela are compared calculating Mortality Rate Ratios for lung cancer through a Poisson regression model, controlling for age and calendar period. The mobility model correctly classifies almost 90% of observations. Its application to the mortality analysis confirms a major risk for lung cancer associated with residence in Gela.

**Conclusions:**

Commuting is a critical aspect of the health-environment relationship in contaminated areas. The proposed methodology can be replicated to different contexts when residential information is lacking or unreliable; however, a careful consideration of the territorial characteristics ("insularity" and its impact on transportation time and costs, in our case) is suggested when specifying the area of application for the mobility analysis.

## Introduction

Epidemiological studies have extensively considered mobility, intended as the change of individual residence, as a risk factor for health. Mobility accounts for accessibility to health services [1-3], and is widely used as a proxy for different times of exposure to environmental pollutants over an individual's lifetime. Furthermore, human mobility has been analyzed as one of the most important vehicles of infectious diseases [4-8]. More generally, the relationship between migration and health is controversial; higher morbidity or mortality rates within in-migration areas, compared with territories showing stable populations, are detected by Boyle et al. [9,10] and Davey Smith et al. [11], in contrast with other evidence [12-15]. Brown and Leyland [16] show that small areas with lower mobility rates in Scotland have better health outcomes, and Martikainen et al. [17] discuss the importance of selective migration when studying the relationship between area socioeconomic characteristics and individual health profiles.

From a methodological perspective, Rogerson and Han [18] argue that mobility may hamper the detection of both geographical differences in disease diffusion and regional variability in disease risk, while Jacquez et al. [19,20] censure "the static world-view in which individuals are considered immobile, migration between populations does not occur, and in which background disease risks under the null hypothesis are assumed to be time-invariant and uniform through geographic space", and propose representation methods of human mobility that account for space-time relations when studying residential epidemiology of long latency illnesses, such as cancer. In their study, "participants must have lived in the study area for at least the past 5 years and had no prior history of cancer".

However, mobility implies not only that people change their residence, but also that (increasingly, with transport facility improvements) they commute on a daily basis, travelling from their residence to a "distant" workplace. From this perspective, commuting has been investigated as a factor that influences individuals' exposure during travel time [21-26], as a source of stress [27] that increases cardio-vascular (CDV) risk [28-30] and travel accident risk [31,32], or as an opportunity to stimulate physical activity both for adult [33-37] and for young people [38-40]. Commuting is also relevant in the identification of different exposure levels to some specific pollutant for populations working within highly polluted areas [41]. This is especially true in the case of "contaminated areas", i.e. areas characterised by the presence of polluting industrial activities; in these sites commuting groups are subject to different exposure periods than resident workers and share "occupational" exposure with their fellows, but have a lower "environmental" exposure than residents, as they live in non-contaminated areas during their non-working time.

Commuting is, then, an important topic in environmental epidemiology and may help to shed light on residential/occupational health risks for different groups. Nevertheless, even though commuting has been intensively studied from geographic (see, among others, [42-44]) and economic perspectives (see Rowendal and Nijkamp [45] for a review of this literature), to our knowledge, there have been no studies attempting to relate commuting and health on the basis of geo-economic models. Therefore, this paper represents an attempt to bridge territorial studies with public health.

With the aim of integrating geo-economic quantitative methodologies with epidemiological studies, this study offers a multidisciplinary perspective in order to improve the definition of groups of exposed populations in cases in which the reconstruction of individuals' residential history is not possible (either due to missing or unreliable information).

Drawing from mobility gravity models, the paper proposes a quantitative method based on the use of a logistic probability model, to define a possible valuable classification of a population of petrochemical workers into commuters and residents categories; on these grounds, we then test residential risk for lung cancer within a cohort of workers from the Gela petrochemical complex (in Sicily, Italy). More generally, the application of commuting analysis may be important for epidemiological investigations, and particularly for occupational epidemiology.

## Background

**Figure 1:**
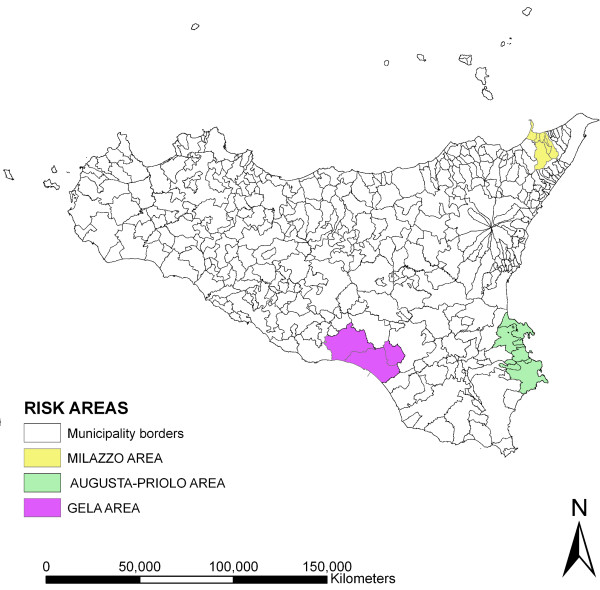
**Sicilian risk areas**.

In Italy, the region of Sicily (which is the biggest island in the Mediterranean) contains three areas that have been declared "at high risk of environmental crisis" ("risk areas"), located in three different portions of the territory, as shown in Figure 1.

Referring to the specific Italian legislation, "risk areas" include territories where the presence of large industrial plants has an important impact on the environment and may harm human health. The general criteria for the determination of the perimeters of risk areas are open to debate and must always be scrutinised. The formal recognition by law of the condition of risk in a particular area, follows specific risk assessment procedures. Sometimes risk areas are identified after the occurrence of particular events raising environmental concern, while in other cases, the recognition of environmental and health risk comes after a long "invisible" process of contamination or when significant epidemiological factors single out a population group living in a particular area [46].

In this study, we specifically concentrate on the site of Gela, a town of 77,000 inhabitants, located on the south-west coast of Sicily and characterised by relative isolation with respect to adjacent municipalities. The town underwent great changes in only a few years after 1956, when oil fields were discovered in its vicinity, both onshore and offshore; since the early 1960s, Gela has hosted a large oil refinery, together with a number of important chemical and petrochemical industries. The industrial area extends along a large portion of the territory in comparison to the size of the urban area.

**Figure 2:**
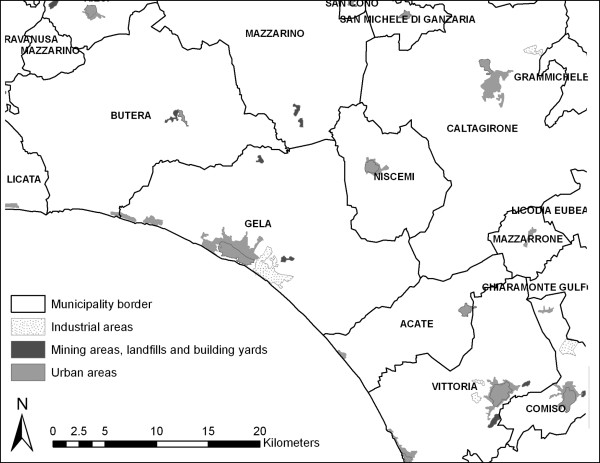
**Gela risk area**.

In 1990, an extended territory comprising the municipalities of Gela, Butera and Niscemi (see Figure 2) was declared an "area at high risk of environmental crisis" (Law n. 349/1986) in consideration both of the serious accidents that may occur at the petrochemical complex and their possible consequences on the local population.

In 1998, a portion of Gela municipality, including the entire petrochemical complex and a vast offshore area, was ruled a "site of national concern for soil remediation" (Law n. 426/1998) due to heavy chemical soil pollution derived from the petrochemical complex.

Several studies report information about environmental contamination levels in the area of Gela; in particular, two specific studies on pine needles and road dust samples showed that the entire area of Gela is heavily affected by industrial and urban emissions of metals and metalloids [47,48]; additionally, data on Gela air quality revealed the presence of high levels of SO_2_, PM_10_, NO_2_, O_3 _and benzene [49], while, as regards the area of concern for soil remediation, underground water and soil are highly contaminated by heavy metals (arsenic, mercury, nickel, manganese, iron, lead, aluminium), hydrocarbons, BTEX (benzene, toluene, ethylbenzene and xylenes) and carcinogenic chlorinated aliphatic compounds [50]. Some of these water and soil pollutants (arsenic, mercury, benzene, nickel) greatly exceed legal limits, thus representing a potential source of health risk for the resident population.

More generally, evidence of a risk of lung cancer, associated with residence near petrochemical plants has been suggested by several studies. A higher risk of lung cancer was observed in people residing in the most polluted areas of Teesside in the UK [51,52] and in case-control studies carried out in the U.S.A., Taiwan and Italy [53-55]. In these studies, exposure to petrochemical industries was indirectly evaluated using the distance of residences from the plants and duration of residence as proxies.

Usually, the contribution of the epidemiological investigation of occupational cohorts is related to the occupational risk evaluated in the production setting under study [56].

In the industrial site of Gela, the large petrochemical complex is the main source of environmental pollution. Some small-area mortality studies of the Gela resident population showed an excessive risk of lung cancer among men and women [57-59], showing also an excessive morbidity risk for acute and chronic diseases of the respiratory system among Gela residents [58,59].

More recent studies have shown that commuting may influence the result of small-area studies and should be carefully considered in the identification of the control population. Selecting areas with similar socio-economic characteristics and with no (or low) commuting to Gela, obtains higher Standardized Mortality Ratios than using neighbouring municipalities [60].

In this frame, the analysis of workers' mortality and/or morbidity profiles can help to elucidate the environmental/residential risk, thus contributing to the description of the local epidemiological context. In fact, a more intense exposure should be assumed for workers residing within the contaminated area compared with commuters, giving rise to the expectation of a higher lung cancer mortality-rate among resident workers.

In general (in Italy as well as in many other countries), information from employment rosters may neither be used to reconstruct residential history, nor to define residence at the moment of enrolment. The latter in particular is true for several reasons: a) workers may be hired before the official change of residence takes place (and there is no need for firms to update their internal documents); b) electoral legislation encourages poll participation by subsidizing travel for people living away from their official place of residence; c) many people may have a strong preference to maintain residence in their birthplace in order to have closer contact with their original family.

In the case of the Gela petrochemical complex, for instance, the employment rosters show that there are workers officially residing at a great distance from Gela, and that Gela's poor communication infrastructures (no motorway, no electrified railway) make it impossible for commuters to reach their workplace, even if they reside in the same region. As a result, residence information from the employment rosters is an unreliable criterion for a qualitative classification of workers as commuters vs. residents, while the simple use of place of birth as a classification criterion, overestimates the number of commuters. Alternative methodologies need to be developed.

Evidence about commuting among workers was initially inferred from several documents and successively confirmed by a qualitative sociological study [61].

Pasetto et al. [62] analyzed mortality within the cohort of petrochemical workers defining "residents" as all workers that were born in Gela or outside Sicily, and "commuters" all workers that were born in another municipality in the same region. This crude classification was due to the lack of a valid criterion to define commuters and raised the problem of the misclassification of residential categories [63,64].

In what follows, a methodology based on the application of mobility models is developed, in order to obtain a more accurate classification criterion to separate "commuting" and "resident" workers, and it is applied to the Gela petrochemical workers' cohort.

## Methods

### Commuting Probability

Mobility is a kind of spatial interaction whose analysis generally relies upon the application of gravity-type schemes [65,66] that consider the quantity of interactions between distant territories as a positive function of their economic force of attraction (or "economic mass"), and as a negative function of the distance that separates them. Economic mass can be expressed using any sound gross measure of economic activity or endowment (local GDP, population, labour force, natural resources, quantity and quality of services per inhabitant, etc.); in the applied literature [67], GDP, population and labour force are the variables most in use to proxy economic mass. Formally, gravitational models for spatial interaction are derived from the Newtonian framework according to the following general formula:

(1) $$T_{ij}=A\left(i\right)B\left(j\right)/F\left(d_{ij}\right)$$

where *T*_*ij *_is the flow of "interactions" between locations *i *and *j*, *A(i) *and *B(j) *are unspecified origin and destination weight functions, which may contain centre attributes relative to its economic mass, and *F(d*_*ij*_*) *is defined as a *distance deterrence *(or *distance-decay*) function, which accounts for the effect of distance *d *on *T*. The specification and combination of origin and destination functions may vary depending on the assumptions made about model parameters and variables. For an in-depth review of the theoretical aspects of gravity models, see Fotheringham and O'Kelly [68].

Gravity models and distance-decay functions have been applied to a wide range of research fields, from the analysis of social phenomena [69,70] to biology and environmental science [71-73], international trade [74,75] urban planning [76] and commuting [67,77,78]. In the field of health and health-care, gravity-type models have been used to build accessibility measures and study spatial accessibility to primary care [79], or to deepen the spatial-temporal dynamics of epidemics in inland towns and coastal cities in England and Wales [80].

As far as the distance-decay function specification is concerned, both the measure of spatial separation and the specific functional form of the relation have been debated [68,81]. For the first aspect, it is possible to use different measures: distance (defined in terms of physical, cultural, social or even religious dimensions), travel cost and travel time. These variables can be combined in various ways [67], but in most applications, physical distance is used as a proxy for both travel cost and travel time [68]. Concerning the formal specification of the distance-decay relation, there is a general consensus about the use of either power or exponential functions (equations (2) and (3)):

(2) $$F\left(d_{ij}\right)=d_{ij}^{-\beta }$$

(3) $$F\left(d_{ij}\right)=\exp\left(-\beta d_{ij}\right)$$

where the parameter *β *measures the effect of distance friction. Structural properties of the exponential function make it appropriate to model short distance interactions (intra-urban mobility), while power specification is more suitable for longer distance interactions such as migration flows [68].

Within an unconstrained setting, the complete model can be structured as follows for power or exponential decay functions, respectively:

(4) $$T_{ij}=v_{i}^{\mu }w_{j}^{\alpha }d_{ij}^{-\beta }$$

(5) $$T_{ij}=v_{i}^{\mu }w_{j}^{\alpha }\exp\left(-\beta d_{ij}\right)$$

From an empirical point of view, a widely used approach to investigate spatial interaction, is to calibrate the model using log-linear regression [81,68]; alternatively, probabilistic models based on Poisson regression [82-84] have been applied.

Our aim in implementing the mobility analysis, is to derive a methodological device that can be used to classify workers into "residents"/"commuters" categories in case the only reliable information is relative to the individuals' birthplace. To this purpose, a gravity-type model is implemented to estimate the probability for a "central locality", where a specific plant is established, to attract commuting flows from other distant places. Probability of commuting will then become the criterion to discriminate observations in the cohort study, according to the workers' place of birth. Working on census data, we need to obtain probabilities from count values, so that a logistic regression that accounts for the origin's economic mass and distance from destination is the most appropriate functional form for this kind of application of gravity-type models.

In our case, we are firstly interested in elucidating to what extent Gela increases commuting flows from "distant" localities in order to identify a critical distance (the "commuting threshold"), discriminating petrochemical workers according to their birthplace. In fact, assuming that Gela-born people employed at the petrochemical complex reside in Gela, workers born outside this municipality may have either moved their residence to Gela, or maintained their residence in a different site, regularly commuting to Gela. Having identified the commuting threshold, workers born outside that distance will be considered "moved to Gela", while individuals born within this area will be classified as "commuters".

Clearly, in the presence of a single destination, its characteristics are invariant in the model: the *B(j) *component of model (1) disappears and mobility can be estimated as an unconstrained function of the origins' economic mass (*A(i)*) and distance from Gela (*d*_*ij*_).

Hence, having transformed Italian Statistics Bureau Census count data on inter-municipal commuting [85] into a dichotomous variable, with value 1 attributed to municipalities with positive commuting towards Gela, and value 0 assigned to municipalities with no commuting to Gela, a logistic regression model has been implemented in order to define the "probability of commuting". The results of this analysis have been applied to classify as "commuting municipalities" all sites whose commuting probability is equal to or greater than 0.5 and "no commuting municipalities" all localities whose commuting probability is lower than 0.5.

The estimated model is then:

(6) $$Ln\left(\frac{p_{i}}{1-p_{i}}\right)=\beta_{0}+\beta_{1}dist+\beta_{2}lf$$

where *p_i _*and 1 - *p_i _*are respectively the probability of observing a positive and a null flow of commuters to Gela. According to the gravitational pattern, explanatory variables are, distance in kilometres (*dist*) of each municipality from Gela and a proxy for the origins' "economic mass", provided by labour force (*lf*); the latter is treated as a dummy variable on the basis of classification into quartiles (see Table 1).

**Table 1:** Labour force classification

lf_1_	Very low (<than 853)
lf_2_	Low (between 853 and 1650)
lf_3_	Medium (between 1650 and 3830)
lf_4_	High (>than 3830) (reference variable)

In order to define the area that has to be considered when implementing the mobility analysis, we propose to identify the longest distance from which a commuter flow is originated and consider this as the array of an ideal circumference, whose area delimitates the boundary of the territorial analysis, excluding all municipalities whose distance from the destination pole is greater than this extension.

In order to limit the extension of our geographic analysis and to define the observations to be considered, we notice that the furthest locality from Gela where a positive commuting is found, is Trapani, at a distance of 248 km.; further, to reach Gela by car from the Italian peninsula, one has to cover a distance of approximately 500 km. at a cost of nearly 100 € per return journey, spending more than 7 hours (data from http://Viamichelin.com), while a one-way journey by train takes from 7 h38 m to 11 h41 m (information from Italian Railways official site). As a consequence, we have restricted the analysis to 367 Sicilian municipalities less than 248 km from Gela.

### Mobility model results

The model is estimated with the maximum likelihood estimator using both the Newton-Raphson and Fisher scoring iterative algorithms; the two methods provide the same results confirming estimates robustness.

As illustrated in Table 2, the result of the Hosmer and Lemeshow Test and the Nagelkerke R^2 ^value (0.66) show a good fit of the estimated probabilities with respect to the empirical ones. The individual significance of coefficients, as measured by the Wald test, is very high, confirming the reliability of the estimated model. Moreover, the signs of coefficients are coherent with the theoretical background.

**Table 2:** Commuting probability estimation results

Dependent variable: 1 = at least one commuter, 0 = otherwise							
						**95% C.I. for Exp(β)**	

	**β**	**Wald**	**Df**	**p-value**	**Exp(β)**	**Lower**	**Upper**

*Dist*	-.040 (.005)	73.291	1	.000	.961	.952	.970
lf*		44.126	3	.000			
*lf(1)*	-2.713 (.502)	29.257	1	.000	.066	.025	.177
*lf(2)*	-2.691 (.474)	32.278	1	.000	.068	.027	.172
*lf(3)*	-2.331 (.469)	24.713	1	.000	.097	.039	.244
*Constant*	6.212 (.724)	73.664	1	.000	498.926		

LR test	243**		4	.000			
Nagelkerke R^2^	.659						
Hosmer and Lemeshow Test	9.709		8	.286			

Standard errors are in parentheses.

*Reference category = High ((>than 3830 units)

** Initial -2LL = 477, Final -2LL = 234.

On the basis of the conventional probability cut-off of 0.5, we assume that no commuting originates from municipalities with a probability value lower than or equal to 0.5, so that, for each "economic mass" category, a threshold distance for commuting can be fixed in correspondence to the 0.5 estimated probability. The model produces four "distance thresholds" relative to the different economic mass consistency of the origin municipalities; these thresholds, as reported in Table 3, define, for each of the envisioned municipality categories, that distance from Gela beyond which commuting is not likely to be observed.

**Table 3:** Distance thresholds

Labour force categories	Distance thresholds (km)
lf_1 _(Very Low labour force)	87.5
lf_2 _(Low labour force)	88
lf_3 _(Medium labour force)	97
lf_4 _(High labour force)	155.3

In accordance with the assumptions of the gravitational model, a greater dimension of the origin municipalities implies higher distance thresholds.

By comparing estimated and observed values, the model correctly classifies nearly 90% of observations (see Table 4).

**Table 4:** Classification decision rule with 0.5 probability cut-off.

Sensitivity	n. of true "commuters"/(n. of true "commuters" + n. of false "no commuters")	74.58%
Specificity	n. of true "no commuters"/(n. of "no commuters" + n. of false "commuters")	92.99%

False Positive Rate	percentage of predicted commuters which are incorrect	17.76%

False Negative Rate	percentage of predicted no commuters which are incorrect	10.64%

Overall percentage correct		87.40%

Table 4 illustrates the model classification decision rule:

As a consequence, we suggest classifying as "commuters" all workers that were born in municipalities lying within the commuting threshold estimated for the mass category, and as "moved to Gela" all those workers who were born outside the threshold.

## Results

### The cohort study

Results of the mobility model were applied to the cohort of Gela petrochemical workers. The following categories of "presumed residence" were identified:

a) *Residents in Gela*: workers born in Gela;

b) *Moved to Gela*when hired: workers born in Sicilian municipalities with the probability of commuting defined by the model as <0.5

c) *Commuters*: workers born in Sicilian municipalities with the probability of commuting defined by the model as ≥ 0.5

The study population included 5,627 workers born in Sicily and employed in the petrochemical complex from 1960 -- the year plant operations started -- to 1993. The vital status follow-up was from 1960 to 2002.

Mortality Rate Ratios (RRs) for all causes, all neoplasms and lung cancer, were estimated comparing workers by residence category. A Poisson regression model, controlling the RR for age and calendar period was applied using STATA 11.0 software; Confidence Intervals, CI (90%) were estimated by the maximum likelihood method. A regression model was also performed adding the variable of job title (results not reported); RR estimates did not significantly change (less than 8% for lung cancer) in the sensitivity analysis made by attributing different job categories to the subjects without information on job title.

Descriptive data on workers by residence category are reported in Table 5.

**Table 5:** Descriptive data on Sicilian workers by residence category

	Commuters	Moved to Gela	Gela
N (workers)	3,234	709	1,684

*Causes of death*	N	N	N

All deaths	342	76	145
Neoplasms	101	23	53
Lung cancer	24	10	20

*Employment & follow-up*	Mean (SD)	Mean (SD)	Mean (SD)

Age at employment	26.1 (5.7)	26.3 (5.4)	26.6 (6.6)
Age at the end of follow-up	58.3 (9.6)	59.7 (9.9)	56.6 (10.2)
Latency^a^	32.2 (9.2)	33.4 (9.7)	30 (9.2)

*Job title*	N (%)	N (%)	N (%)

Blue collar	1,665 (51.5)	253 (37.7)	1,067 (63.4)
White collar	727 (22.5)	263 (37.1)	188 (11.2)
Both	608 (18.8)	142 (20)	322 (19.1)
Missing	234 (7.2)	51 (7.2)	107 (6.3)

^a^Period from hiring to the end of follow-up (duration of follow-up.)

Comparing mortality by place of birth and using *commuters *as a reference, the RR for lung cancer is 1.71 (0.92-3.17) for workers classified as *moved to Gela*, and 1.7 (1.03-2.81) for workers born in Gela; results are reported in Table 6.

**Table 6:** Rate Ratio (RR) of mortality from all causes, all neoplasms and lung cancer by residential categories using *commuters *as a reference

**Cause of death (IX ICD**^**a**^**)**	Category of residence	**RR**^**b**^	90% CI
All causes (001-999)	Commuters	1.0	-
	Moved to Gela	0.9	0.73-1.11
	Residents in Gela	0.88	0.75-1.03

All neoplasms (140-208)	Commuters	1.0	-
	Moved to Gela	0.94	0.64-1.37
	Residents in Gela	1.09	0.82-1.44

Lung cancer (162)	Commuters	1.0	-
	Moved to Gela	1.71	0.92-3.17
	Residents in Gela	1.7	1.03-2.81

^a ^International Classification of Disease IX revision codes

^b ^RR adjusted for age and calendar period

The results obtained by applying the proposed categorization of residence are coherent with our hypothesis on residential risk for lung cancer mortality: a major risk is associated with residence in Gela.

RR results could be partly attributed to differences in distribution of other risk factors for lung cancer between residential categories, such as differences in smoking habits, which is the main recognized risk factor for lung cancer [86]. Though, examples of substantial confounding are rare in occupational epidemiology [64].

Despite some limitations, the results support the hypothesis that *commuters *have a lower residential/environmental risk of mortality from lung cancer compared with workers who were likely to have been residents in Gela.

## Discussion and Conclusions

In this paper a multiple logistic regression model has been implemented for the estimation of distance thresholds to be applied in order to classify Gela's petrochemical workers in the following categories: "commuters", "moved to" and "residents". As in many retrospective studies, information from employment rosters about workers' place of residence are not reliable and reconstruction of residential history is not possible.

In similar situations, we suggest classifying population according to birthplace information, implementing the following procedure: a) using external data on workers' mobility (e.g., census data), construct a dichotomous variable for municipalities according to the presence of workers commuting to the destination under study (Gela, in our case), attributing value 1 in the case of positive mobility and value 0 in the case of no commuters; b) classify municipalities according to the dimension of their labour force, grouping count data into quartiles in order to consider the role of "economic mass" of localities; c) implement a logistic regression model to estimate a "probability of commuting" as a function both of distance and of four dummies accounting for the "economic mass" of the origin municipality; d) define "commuting origins" all municipalities where the probability of commuting is equal to or higher than 0.5 and, consequently, establish the "commuting threshold" at the distance that corresponds to a probability of 0.5 for each dimensional group of municipalities; e) classify workers that were born outside the commuting threshold as "moved to", workers that were born within the threshold as "commuters", and workers that were born in the destination municipality as "residents"; f) analyze data according to the resulting classification.

As far as the mobility analysis is concerned, the study is subject to implicit assumptions and restrictions:

1) The cohort study regards a specific group of workers (petrochemical workers), while mobility analysis is conducted on the labour force as a whole. This implies that petrochemical workers have the same commuting behaviour as the total Gela labour force.

2) The cohort study refers to the population that had been hired at the petrochemical complex during the 1960-1993 period, while the mobility analysis was based upon 2001 census observations. This implicitly assumes that the commuting habits of the Gela labour force (and of the petrochemical cohort) remained constant over the period 1960-2001.

3) Mobility analysis does not account for differences in accessibility conditions between the origin municipalities. Further developments should consider the distinction between different road characteristics (principal and secondary) and control for other modal conditions (railways).

The definition of residential categories obtained from the analysis of the observed mobility patterns, reduces misclassification of residential status for a qualitative categorisation, in the absence of the individual data necessary to correctly define a residential history for each subject. Further research should reveal more about the impact of commuting on exposure to environmental risk factors for populations living/working within contaminated areas.

Finally, as no ad hoc assumption is required, the methodology can be replicated to different contexts. Verification of mobility phenomena using qualitative sociological studies is suggested. Furthermore, a careful consideration of the specific characteristics of the regional context ("insularity" and its impact on transportation time and costs, in our case) is advised when specifying the area of application for the mobility analysis.

## Conflict of interests

The authors declare that they have no competing interests.

## Authors' contributions

GS collaborated on the development of the idea for the study, on the definition of the mobility model supervising the research; RPo undertook the mortality analysis providing the workers' cohort data and the results section; EG worked on data acquisition and contributed to the mobility analysis providing the commuting probability sub-section; MM performed the statistical analysis for specifying commuting probabilities and prepared the mobility model results sub-section; MLR provided feedback on commuting study design and interpretation of results. PM cooperated to the background analysis as far as the study area is concerned, providing figures and maps. All authors read and approved the final manuscript.
